# Exploring ORFan Domains in Giant Viruses: Structure of Mimivirus Sulfhydryl Oxidase R596

**DOI:** 10.1371/journal.pone.0050649

**Published:** 2012-11-28

**Authors:** Motti Hakim, Daria Ezerina, Assaf Alon, Ohad Vonshak, Deborah Fass

**Affiliations:** Department of Structural Biology, Weizmann Institute of Science, Rehovot, Israel; Research Center Borstel, Germany

## Abstract

The mimivirus genome contains many genes that lack homologs in the sequence database and are thus known as ORFans. In addition, mimivirus genes that encode proteins belonging to known fold families are in some cases fused to domain-sized segments that cannot be classified. One such ORFan region is present in the mimivirus enzyme R596, a member of the Erv family of sulfhydryl oxidases. We determined the structure of a variant of full-length R596 and observed that the carboxy-terminal region of R596 assumes a folded, compact domain, demonstrating that these ORFan segments can be stable structural units. Moreover, the R596 ORFan domain fold is novel, hinting at the potential wealth of protein structural innovation yet to be discovered in large double-stranded DNA viruses. In the context of the R596 dimer, the ORFan domain contributes to formation of a broad cleft enriched with exposed aromatic groups and basic side chains, which may function in binding target proteins or localization of the enzyme within the virus factory or virions. Finally, we find evidence for an intermolecular dithiol/disulfide relay within the mimivirus R596 dimer, the first such extended, intersubunit redox-active site identified in a viral sulfhydryl oxidase.

## Introduction

More than half the approximately 900 predicted protein-coding genes in *Acanthamoeba polyphaga* mimivirus are ORFans [1], *i.e*., they have no detectable homologs in sequence databases and thus no predicted functions [2]. Many ORFan proteins, or proteins containing ORFan domains, are incorporated into mimivirus virions, indicating that they are indeed expressed [3]. The origin of ORFan proteins and domains is poorly understood. It remains to be determined how many ORFans correspond to known folds but fall below the threshold for detection on the basis of sequence homology or fold recognition, *versus* how many truly represent novel structural units. Data addressing this question may help determine whether mimivirus and other nucleocytoplasmic large DNA viruses (NCLDVs) exhibit so many apparent ORFans due to rapid sequence divergence or through a mechanism for generating novel folds.

Mimivirus R596 is an enzyme of the Erv family of sulfhydryl oxidases, which catalyze the formation of disulfide bonds using an active-site di-cysteine motif juxtaposed to a flavin adenine dinucleotide (FAD) cofactor [4]. Erv disulfide catalysts are apparently universal in eukaryotic species and are a highly conserved element of NCLDVs as well. Erv enzymes, by definition, share the same fold, co-factor binding capability, and active-site cysteines. They differ, however, in features outside this catalytic core. For example, most cellular Erv enzyme FAD-binding domains are flanked by regions of polypeptide known or predicted to be disordered. Typically found within these flanking regions is a second redox-active cysteine pair, which interacts with substrate and transfers electrons to the FAD-proximal disulfide [5]–[6].

Viral Erv enzymes show particularly great diversity outside the FAD-binding, catalytic core. Many viral Erv sulfhydryl oxidases are highly compact, lack a shuttle disulfide, and contain only the single disulfide adjacent to the FAD. Other viral disulfide catalysts, however, have additional domain-sized segments fused to the Erv module. For example, the baculovirus sulfhydryl oxidase, Ac92, contains an amino-terminal fusion with a folded structure, but this domain does not seem to have a redox role in catalysis [7]. Mimivirus, ascovirus, and nudivirus sulfhydryl oxidases contain carboxy-terminal fusions, which bear no resemblance to any known protein or domain and thus may be considered ORFan regions [8]. In these cases, the structural or functional role of these extensions in the context of sulfhydryl oxidase activity remains to be determined. The mimivirus sulfhydryl oxidase, in particular, has cysteine residues within its ORFan region that may participate in structural or redox-active disulfides. For comparison, the Erv-related cellular enzyme known as quiescin sulfhydryl oxidase (QSOX) has an additional redox-active domain tethered to the FAD-binding domain, and the former makes essential mechanistic contributions to the catalysis of disulfide formation [9]. As viral Erv sulfhydryl oxidases are more divergent than their cellular counterparts, mechanistic studies of these viral enzymes may reveal new types of cysteine-based electron relays.

X-ray crystallographic studies of viral enzymes that catalyze disulfide bond formation have already revealed diversity in structure and assembly beyond that seen in cellular enzymes of the same family. Specifically, the African swine fever virus sulfhydryl oxidase, pB119L, and baculovirus Ac92 both use orthogonal protein surfaces for dimerization compared to cellular Erv sulfhydryl oxidases. In fact, the pB119L and Ac92 dimerization interfaces are orthogonal to one another as well [7], [10]. Here we present the structure of the intact mimivirus sulfhydryl oxidase R596, including both its sulfhydryl oxidase domain and its ORFan domain. Mimivirus R596 is one of the largest viral Erv polypeptides found to date in viral genomes, forming a dimer of ∼69 kD. Some similarities in overall shape and surface properties may be noted between mimivirus R596 and baculovirus Ac92, but in general the mimivirus R596 structure is a remarkable new variation within the Erv enzyme family. It is also the first viral sulfhydryl oxidase to be characterized that appears, based on results presented herein, to function with the aid of a shuttle disulfide, albeit within a sequence motif different from those described previously for cellular Erv enzymes [5].

## Results

### Preparation of Mimivirus R596 for Crystallization

Recombinant, full-length R596 purified from bacteria migrated by SDS-PAGE as various high molecular weight species under non-reducing conditions, but as a single band of the expected size under reducing conditions. Moreover, the purified protein continued to oxidize and to precipitate from solution. This phenomenon occurred also when the enzyme was produced in the Origami™ B *E. coli* strain, which is deficient in cytoplasmic thiol reductases and thus suited to disulfide bond formation in the cell cytoplasm.

**Figure 1:**
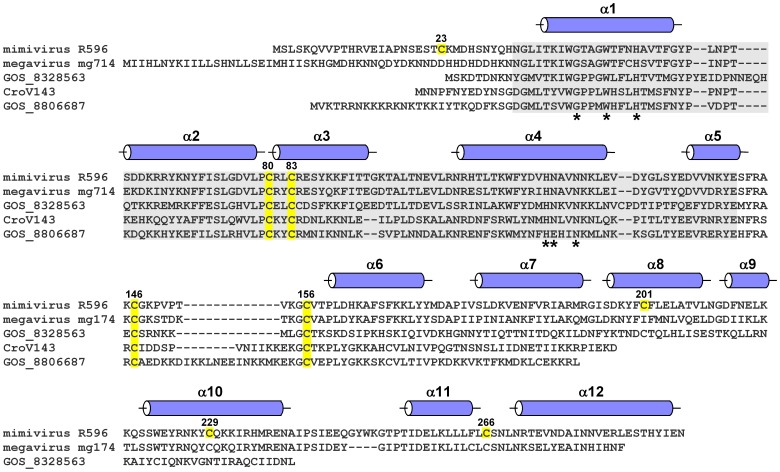
Amino acid sequence alignments of putative viral sulfhydryl oxidases related to mimivirus R596. Cysteine residues in R596 are highlighted in yellow and numbered according to amino acid position. The four cysteines shared in all aligned proteins are highlighted in all the sequences. The gray background indicates the region corresponding to the Erv family FAD-binding fold. Asterisks below columns in the alignment indicate conserved positions in the FAD-binding site. The positions of helices, according to the R596-4C structure, are shown as cylinders above the R596 sequence. Sequence accession numbers are: mimivirus R596: Acanthamoeba polyphaga mimivirus (YP_003987112); megavirus mg714: Megavirus chiliensis (YP_004894765); GOS_8328563: marine metagenome (EBM93594.1); CroV143: Cafeteria roenbergensis virus BV-PW1 (YP_003969775); GOS_8806687: marine metagenome (EBJ98940.1).

We inspected the cysteine residues in the R596 sequence to identify the cause of covalent aggregation. The eight R596 cysteines are positioned as follows: the first is upstream of the Erv domain, two more constitute the active site of the Erv domain, and the last five are downstream of the canonical Erv fold (Figure 1). The roles of the six cysteines outside the Erv domain were unknown. Comparing the R596 orthologs in *Cafeteria roenbergensis* virus (CroV) [11], *Megavirus chilensis* [12], and a few additional sequences likely to have arisen from uncharacterized viruses, we observed that the two cysteines immediately downstream of the Erv domain are the most highly conserved aside from the active-site disulfide (Figure 1). A new mimivirus R596 construct (R596-4C) was designed to contain only the four conserved cysteines: Cys80, Cys83, Cys146, and Cys156. R596-4C remained soluble throughout purification and later yielded diffracting crystals.

### Structure of Mimivirus R596

Mimivirus R596-4C was crystallized in space group P6_1_22 with one protomer in the asymmetric unit. Diffraction data were collected to 2.21 Å resolution. Phases were determined by multi-wavelength anomalous dispersion using a crystal of R596-4C substituted with selenomethionine. Data collection and refinement statistics are in Table 1. The structure model spans R596 residues 32–148 and 157–292. No interpretable electron density was observed for residues 1–31 and 149–156, and the density for residues 145–148 and 157–159 was poor. Though the crystal asymmetric unit contained only one polypeptide, application of crystallographic symmetry operators generated the dimer structure.

**Table 1:** Data collection, phasing, and refinement statistics for mimivirus R596-4C.

	Native		SeMet	
**Data collection**				
Space group	**P6_1_22**		**P6_1_22**	
Cell dimensions				
*a*, *b*, *c* (Å)	91.3, 91.3, 200.8		91.2, 91.2, 199.6	
α, β, γ (°)	90, 90, 120		90, 90, 120	
		*Peak*	*Inflection*	*Remote*
Wavelength (Å)	0.9766	0.97887	0.97898	0.97660
Resolution (Å)	45−2.21 (2.33−2.21)	45−2.21 (2.32−2.21)	45−2.21 (2.32−2.21)	45−2.21 (2.32−2.21)
R_sym_ a	0.094 (0.742)	0.094 (0.767)	0.082 (0.556)	0.126 (0.372)
*I/*σ	18.9 (4.4)	29.2 (4.3)	30.8 (5)	29.5 (5)
Completeness (%)	97.2 (93.5)	99.9 (99.3)	99.9 (99.6)	99.6 (97.3)
Redundancy	19.3 (19.2)	35 (20.6)	35.4 (21.7)	38.4 (38)
**Overall figure of merit** b		0.42/0.67		
**Refinement**				
Resolution (Å)	50−2.21			
No. reflections/test	23,329/1718			
R_work_/R_free_ c	0.228/0.254			
No. atoms				
Protein	2103			
FAD	53			
Water	122			
R.m.s deviations				
Bond lengths (Å)	0.007			
Bond angles (°)	1.08			

Values in parentheses are for the highest-resolution shell.

a R_sym_ = Σ*_hkl_*Σ*_i_*|*I_i_*(*hkl*) - <*I*(*hkl*)>|/Σ*_hkl_*Σ*_i_I_i_*(*hkl*), where *I_i_*(*hkl*) is the observed intensity and <*I*(*hkl*)> is the average intensity for *i* observations.

b Figures of merit obtained from Phenix (31).

c R_work_, R_free_ = Σ||*F*_obs_| - |*F*_calc_||/Σ|*F*_obs_|, where *F*_obs_ and *F*_calc_ are the observed and calculated structure factors, respectively. A set of reflections (7.4%) were excluded from refinement and used to calculate R_free_.

**Figure 2:**
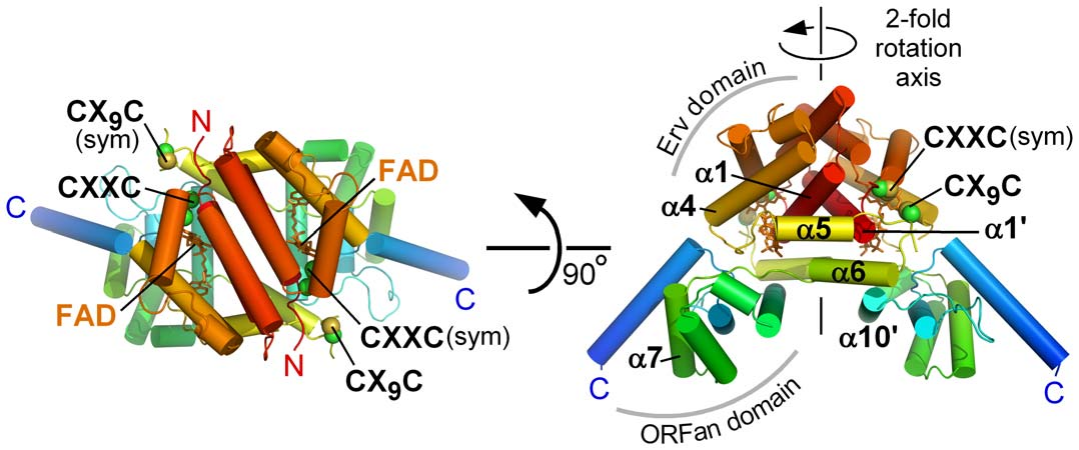
Structure of mimivirus R596-4C. The R596-4C dimer is shown in orthogonal orientations. The left panel is viewed down the two-fold rotation axis. In the right panel, the two-fold rotation axis is vertical in the plane of the page. Helices are represented as cylinders and colored in a gradient from red (amino terminus; N) to blue (carboxy terminus; C). The FAD cofactor is shown as orange sticks. Cysteine side chains are shown as spheres (Cβ, green; S, gold). The FAD-proximal, active-site disulfide is labeled “CXXC.” The second cysteine pair in R596-4C is labeled “CX_9_C.” The designation “(sym)” indicates that the disulfide is from the symmetry-related subunit. Certain secondary structure elements are labeled, and a tag (’) indicates an element in the second subunit.

The R596-4C structure is highly helical (Figure 2) and has a two-domain architecture. The structure of the amino-terminal, FAD-binding domain of R596, R596_Erv_, was determined previously [10]. Only minor differences, primarily in side chain orientation, were found between the R596_Erv_ structure determined alone and within R596-4C. These differences are concentrated in the first (α1) and last (α5) helices of the Erv domain. Surprisingly, these variations arise because the next helix, α6, which was anticipated to be part of the ORFan domain, interacts primarily with the Erv domain instead. Rather than clustering spatially with the carboxy-terminal helices of R596, helix α6 packs against helix α5 from its own chain and helix α1 from the Erv domain of the other protomer (α1’) (Figure 2). The presence of helix α6 displaces Lys38 and Phe142 in helices α1’ and α5, respectively, to avoid steric clashes.

Although helix α6 is more closely associated with the Erv rather than the ORFan domain, it nevertheless contacts the ORFan domain of the symmetry-related subunit of the R596-4C dimer, at helix α10’, and thereby contributes to the quaternary structural assembly of this enzyme. The tightly intertwined R596-4C dimer (Figure 2) has a larger buried surface area (1150 Å^2^) than the R596_Erv_ dimer alone (680 Å^2^). As a major component of this extended interface, α6 buries the hydrophobic residues Leu160, Ala164, Phe167, Leu170, and Tyr171 against helices α1’ and α10’.

**Figure 3:**
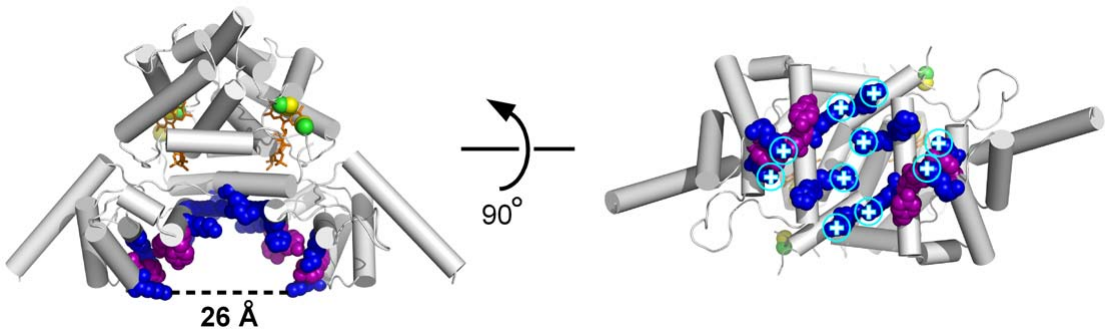
Character of the concave face of R596-4C. Exposed basic and aromatic residues in the groove between the ORFan domains are shown in space-filling representation. Phenylalanine and tyrosine residues are purple; arginine, lysine, and histidine are blue. In the view into the groove (right), arginine and lysine side chains are marked with a circled + symbol.

The ORFan domain contributes to the expanded dimer interface only by contacts with the Erv domain from the second subunit of the dimer and not with its own symmetry-related domain. The two ORFan domains in the dimer form the sides of a groove, with α10 and the amino-terminal ends of α8 and α11, together with α8’, α10’, and α11’, lining the inner walls of the groove. The groove walls and base have an overall basic charge and a number of exposed aromatic groups (Figure 3), representing possible specific interaction sites with other macromolecules.

### The Mimivirus R596 ORFan Domain

**Figure 4:**
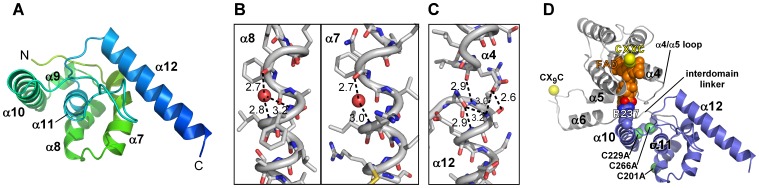
The R596-4C ORFan domain. (A) A single R596-4C_ORFan_ domain is shown with secondary structure elements labeled. (B) Two irregular R596-4C_ORFan_ domain helices with water-mediated i-to-i+4 hydrogen bonding interactions. The water oxygen atom is shown at half its van der Waals radius. Dashed lines are labeled with distances (Ångstrom) between hydrogen bond donor and acceptor. (C) The carboxy-terminus of helix α4 in the Erv domain caps helix α12 in the ORFan domain. Inter- and intrahelical hydrogen bond donor-acceptor distances (Ångstrom) are indicated. (D) R596 interdomain interface. One subunit of R596-4C is shown with the Erv domain in gray and the ORFan domain in slate blue. Cβ atoms at sites of cysteine-to-alanine mutations are shown as lime-green spheres and marked by residue number. Extended segments of polypeptide sandwiched in the interdomain interface are labeled (α4/α5 loop and interdomain linker). The FAD is in orange, space-filling representation, with the O1P and O2P atoms distinguished in red. Arg237 from the ORFan domain, which approaches the FAD at O1P/O2P, is shown in space-filling representation. The lack of participation of helices in domain-domain packing is evident.

The R596-4C ORFan domain (R596-4C_ORFan_, residues 180–292) is composed of six helices, α7 through α12 (Figure 4A). A structure similarity search [13] found no close match for R596-4C_ORFan_, suggesting that it represents a new fold. The highest scoring protein, with a Z-score of 3.6, gave a root mean square deviation in Cα positions of 3.7 Å with only 64 residues included in the alignment and does not appear to be significant. The R596-4C_ORFan_ fold consists of a short, central, hydrophobic helix (α11) surrounded by three other helices (helices α7, α8 and α10). Another small helix (α9) closes off the bottom of the helical bundle, when the bundle is viewed down helix α11 (Figure 4A). Projecting out from the helical bundle is the final, carboxy-terminal helix (α12). Residue 201, a cysteine mutated to alanine in R596-4C, is exposed to solvent on the face of helix α8. Cys201, along with Cys23 in the disordered amino-terminal region, may have contributed to the aggregation of wild-type R596. In contrast, residues 229 and 266, which are cysteines in wild-type R596 but alanines in R596-4C, are buried in the R596-4C_ORFan_ core and likely form a disulfide bond in the wild-type protein. These two buried cysteines are also found in the megavirus R596 homolog, mg174 (Figure 1).

The R596-4C_ORFan_ domain has a number of notable geometric features and capping solutions for its helices. For example, helices α7 and α8 are kinked due to a water-bridged C = O^…^H-N hydrogen bond in each (Figure 4B). Helix α7 has three β-branched residues, considered to be poor helix-formers [14], and is not predicted to be helical by PSIPRED [15]. In helix α8, the single turn of helix amino-terminal to the break contains a lysine-tyrosine pair, a common feature at the carboxy-termini of helices [16]. In fact, Tyr199 in α8 is sandwiched between two lysines in an apparent extended cation-π interaction. Regarding helix capping, a few of the R596-4C_ORFan_ helices are capped with assistance from the R596-4C_Erv_ domain. In particular, α12 is capped both by an asparagine at its own N-cap position and by a glutamate side chain and backbone carbonyl from the carboxy terminus of α4 in the R596-4C_Erv_ domain (Figure 4C). Additionally, the carboxy terminus of α10 is capped by a lysine (Lys168) from α6 of the symmetry-related molecule. These and other interactions, described below, lead to an intimate but somewhat unusual interface between the two domains within each protomer and between the two symmetry-related subunits of the dimer.

### Interaction of the R596 Erv and ORFan Domains

Remarkably, though both R596 domains are highly helical, helix-helix interactions generally do not contribute to the association of the domains. The exception to this generalization is the end-on interaction between helices α4 and α12 as described above and shown in Figure 4C. Instead, the R596-4C_ORFan_ domain interacts with the R596-4C_Erv_ domain primarily *via* regions lacking well-defined secondary structure. In particular, the primary participants in the association of the Erv and ORFan domains within one molecule are the loop connecting Erv domain helices α4 and α5 and the extended segment between helix α6 and the ORFan domain (Figure 4D). Furthermore, the interdomain interface includes parts of the FAD cofactor that are solvent-exposed in most Erv enzymes. For example, Arg237 projects from α10 and interacts with an FAD phosphate group (Figure 4D). Hydrogen bonds are the dominant interactions in the interface between the two domains in one R596 protomer, and further stabilization is apparently provided by a salt bridge between Asp134 (helix α5) and Lys217 (helix α9). Overall, the inter-domain linker (residues 171–177) is sandwiched between the two domains and makes polar interactions with the exposed FAD in the Erv domain and helix α10 from the ORFan domain. In addition, several hydrophobic residues from the linker (Ala175, Ile177, and Val178) contribute to the ORFan domain hydrophobic core, though the linker itself drapes over the domain and is not deeply embedded within it.

#### Mimivirus R596 contains multiple disulfides that contribute to catalysis

R596-4C was tested for its ability to oxidize various model substrates. It showed sulfhydryl oxidase activity on the reducing agents dithiothreitol (DTT) and tris(hydroxymethyl)phosphine (THP) but no detectable activity on the protein substrates reduced thioredoxin or reduced and denatured RNase A. DTT was used as the substrate for further enzymatic characterization of R596.

**Figure 5:**
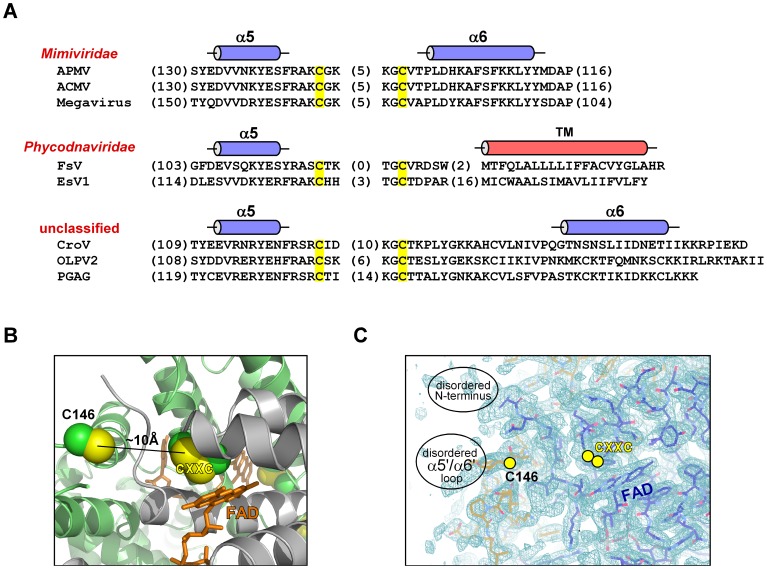
Putative shuttle disulfide in viral sulfhydryl oxidases. (A) Amino acid sequences spanning the putative shuttle disulfide in the sulfhydryl oxidases of mimivirus and related viruses. In parentheses are the numbers of amino acids in the indicated locations not explicitly represented in the alignment. Observed or predicted helices are shown as cylinders. (B) The active-site region of the R596-4C dimer is shown with one subunit gray and the second lime green. The FAD is in orange sticks. The approximate distance between the position of the C146–C156 disulfide and the FAD-proximal cysteines is indicated, with the caveat that C146 is on the edge of a region with extremely poor electron density and its orientation cannot be taken as definitive. (C) 2Fo-Fc electron density contoured at 1.4σ is shown at the protein-solvent boundary, indicating the approximate positions of the disordered amino-terminal region of the protein and the loop bearing the C146–C156 disulfide. Cys156 is not visible in the electron density map, and the position of Cys146 should be taken as approximate.

**Figure 6:**
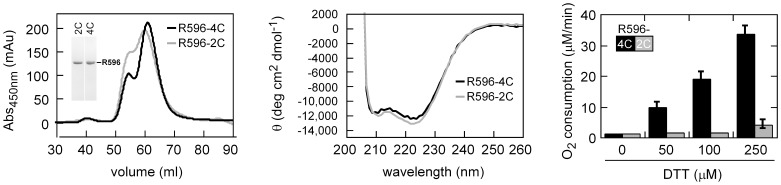
Contribution of C146/C156 to catalysis. (A) Gel filtration and gel electrophoretic (inset) profiles and (B) circular dichroism spectra of R596 variants containing (R596-4C) and lacking (R596-2C) the C146/C156 cysteine pair. These experiments demonstrate that both variants are intact and have similar secondary and quaternary structures. The R596 variants show two overlapping peaks by gel filtration. This phenomenon is not due to protein degradation, and both peaks of R596-4C gave similar catalytic activity (not shown). (C) Initial rates of oxygen consumption by R596 variants (1 µM) were monitored after addition of DTT at the indicated concentrations. The R596 variant containing the apparent shuttle disulfide, R596-4C, is considerably more active (black bars) than the variant, R596-2C, in which the C146 and C156 cysteines were replaced by alanines (gray bars). The R596-4C values are the average and standard deviation of at least four measurements from two independent preparations of enzyme.

A notable feature in mimivirus R596 and other related viral sequences is the di-cysteine signature downstream of helix α5 (Figures 1,5A). In the R596-4C structure, these cysteines, Cys146 and Cys156, appear to be within 10 Å of the FAD-proximal cysteine pair, Cys 80 and Cys83, from the symmetry-related subunit (Figure 5B, left). This position is consistent with the possibility that Cys146 and Cys156 function as a shuttle disulfide similar to those found in cellular Erv enzymes [5]–[6]. Notably, the lack of clear electron density seen for Cys146, Cys156, and the intervening loop in the R596-4C crystal structure (Figure 5B, right) implies that the loop may be sufficiently mobile to approach the FAD-proximal cysteines. Poor or absent electron density was previously observed for shuttle disulfides during determination of crystal structures of cellular Erv enzymes [5], [17]. However, shuttle disulfides in cellular Erv enzymes are found in the motifs Cys-X-Cys, Cys-X-X-Cys, or Cys-X_4_-Cys [5]–[6], [17]–[18], whereas a Cys146-Cys156 disulfide in mimivirus R596 would close a larger ring (CX_9_C). To test whether this disulfide is nevertheless a functional shuttle that contributes to catalysis [17]–[18], a R596-4C variant was constructed in which Cys146 and Cys156 were mutated to alanine. This variant, R596-2C, which retains only the two cysteines of the FAD-proximal disulfide, was produced with high yields using the same protocol as for R596-4C and was shown to be structurally intact (Fig. 6A,B). R596-4C was considerably more active than R596-2C in DTT oxidation (Figure 6C), suggesting that Cys146 and Cys156 indeed have a functional role in catalysis.

## Discussion

NCLDVs have enormous genomes compared to other viruses, high mutation rates compared to cell-based organisms, and vast population sizes. They may also exhibit intense competition for hosts. These combined features imply that protein sequence space may be sampled and selected more aggressively by NCLDVs than by any other group of viruses with the possible exception of bacteriophage.

We have used the NCLDV sulfhydryl oxidase, one of the core proteins shared by diverse NCLDVs, for a case study of protein structural diversity in NCLDVs and between NCLDVs and eukaryotes. We previously observed that the manner in which the FAD-binding fold of the viral sulfhydryl oxidases assembles into quaternary structures varies widely among NCLDVs and other large viruses [7], [10]. In the current study, we show that viral sulfhydryl oxidases exhibit tertiary as well as quaternary structural innovation. The mimivirus R596 enzyme contains an additional domain not found in other sulfhydryl oxidases from either cells or viruses, and this domain does not share detectable sequence homology with any other known protein. Upon solving the R596 structure, we could conclude that this additional domain has a novel tertiary structure, as automated protein structure comparison [13] places it far from other entries in the protein structure database.

The sequences of putative related viral sulfhydryl oxidases, displayed in Figure 1, can also be interpreted in light of the R596 ORFan domain structure. The megavirus mg174 sequence shows sequence homology spanning all helices of the mimivirus R596 structure, suggesting that it shares the ORFan domain fold, albeit with a shorter final helix. In contrast, the global ocean sampling sequences GOS_8328563 and GOS_8806687, as well as the putative CroV sulfhydryl oxidase, do not seem to contain the full helix complement. In particular, helix α11 is the central helix in the R596 ORFan domain fold, so any polypeptide assuming this fold would be expected to have a clear counterpart to α11, which cannot be identified in the amino acid sequences of GOS_8328563, GOS_8806687, or CroV gene product 143. These other putative R596 orthologs may have alternative, more diminutive folds downstream of helix α6. Alternatively, their downstream sequences may be structurally disordered or may fold in association with their respective Erv domains.

In addition to a domain with a novel fold, the mimivirus sulfhydryl oxidase R596 appears to contain a new variety of shuttle disulfide. The shuttle disulfide of sulfhydryl oxidases is typically found on a flexible loop or tail of the enzyme, accepts two electrons during substrate oxidation, and passes these electrons to the active-site disulfide adjacent to the FAD or other cofactor. A shuttle disulfide has not been observed in other viral sulfhydryl oxidases to date. We have demonstrated that mimivirus R596 contains a cysteine pair from one subunit of the dimer located in the vicinity of the active-site disulfide of the second subunit. This location is consistent with the position of a shuttle disulfide, as observed in the fungal endoplasmic reticulum Erv2 protein [5] and mitochondrial Erv1 enzymes [6], [17]. However, the CX_9_C motif for a shuttle disulfide has not been observed previously. This inter-cysteine spacing is conserved in mimivirus isolates, but phycodnaviruses and some unclassified viruses show various di-cysteine motifs, ranging from CX_4_C to CX_18_C (Figure 5A).

Our experiments demonstrate that the mimivirus sulfhydryl oxidase R596 is much more active with the CX_9_C cysteines present than when this motif is mutated to AX_9_A. The most straightforward explanation for this observation is that DTT readily reduces the CX_9_C motif, which in turn readily reduces the FAD-proximal, active-site disulfide, and that this series of events occurs faster than DTT reduces the active-site disulfide directly. Indeed, in some enzymes containing the Erv domain, the presence of an additional disulfide distal from the FAD greatly enhances the rate of oxidation of DTT according to the shuttle mechanism [20]–[22], whereas in other Erv enzymes, the small and highly reducing DTT reagent bypasses the shuttle disulfide and reduces the active-site disulfide [17]. Mimivirus R596 appears to be of the former type. It cannot yet be ruled out, however, that the R596 CX_9_C cysteines instead have an activating role but do not participate directly in electron transfer. It is also conceivable that the CX_9_C motif plays a currently under-appreciated structural role, despite the flexibility of this region as suggested by the R596-4C electron density maps (Figure 5B, right). In this case, the R596-2C mutant would be poorly active due to undermining the structural foundations of the FAD-proximal, active-site chemical environment rather than due to direct participation of the CX_9_C motif in catalysis. We do not consider this possibility very likely, however, as the R596 Erv domain was produced and crystallized in isolation [10], demonstrating its structural independence from the entire ORFan domain, and hence from the ORFan domain cysteines.

If the R596 CX_9_C motif does indeed function as a shuttle disulfide, accepting electrons from substrate cysteines and relaying them to the FAD-proximal disulfide, the CX_9_C disulfide would be the primary determinant of substrate specificity. We observed that R596-4C failed to oxidize two model reduced protein substrates, one unfolded (RNase A) and the other folded (thioredoxin). This observation is not unexpected, as Erv family sulfhydryl oxidases studied previously showed widely different activities on model protein substrates. For example, *A. thaliana* Erv1 oxidizes reduced thioredoxin, whereas *S. cerevisiae* Erv2 does not [17]. In many cases, as for R596, the physiological targets of enzymes that catalyze disulfide bond formation are not yet known. Investigation of the activity of these enzymes on non-physiological protein or small molecule model substrates can be highly informative regarding reaction mechanisms and routes of internal electron transfer. Nevertheless, these experiments should not be interpreted as definitive regarding the potential for these enzymes to oxidize other proteins in other environments. The issue of enzyme environment is highly relevant to viral sulfhydryl oxidases, which, at least in some cases, are localized to viral factories and incorporated into virions [23]–[25]. The associations involved in enzyme localization may influence activity by allosteric mechanisms or by increasing proximity to substrate pools.

Along these lines, and given the shape and surface properties of full-length R596, it is possible that the ORFan domain provides protein-protein or protein-nucleic acid interaction sites to support the localization or substrate selection of the catalytic domain. For example, the dimensions and charge of the prominent cavity in the R596 dimer are consistent with the possibility that the enzyme straddles a DNA or RNA double helix. However, R596 does not appear to have α-helices appropriately positioned for insertion into DNA major grooves, and the issue of nucleic acid binding remains an open question. R596 transcript is present at low levels in virus-infected cells [19], and protein levels may be correspondingly low, so targeting of the sulfhydryl oxidase to its proper microenvironment in viral factories or assembling mimivirus virions may be critical for R596 function. Based on the prevalence of apparent ankyrin repeats, the majority of accessory domains in mimivirus proteins are likely to be involved in modulating the protein-protein interaction network that underlies viral takeover of infected cells. Nevertheless, it will be interesting to determine whether, among other ORFan domains of mimivirus proteins, novel catalytic activities have also arisen.

## Materials and Methods

### Mimivirus R596 Production and Purification

The R596 open reading frame was amplified by PCR from *Acanthamoeba polyphaga* mimivirus genomic DNA (provided by Abraham Minsky, Weizmann Institute of Science) and inserted into the pET-15b vector (Novagen) between the NdeI and BamHI restriction sites. The R596-4C variant with Cys23, Cys201, Cys229 and Cys266 residues mutated to alanine was generated by restriction-free cloning [26]. Protein expression was done in *E. coli* strain BL21 (DE3). Transformed cells were grown in LB containing 100 mg/l ampicillin to an OD_595 nm_ of 0.5–0.6 at 37°C. Isopropyl-1-thio-β-D-galactopyranoside was added (0.5 mM), and the cultures were grown for a further 48 hr at 15°C. Cells were harvested and suspended in 40 mM sodium phosphate buffer, 400 mM NaCl, 20 mM imidazole, and 20% glycerol (pH 8.0), sonicated on ice, and centrifuged for 40 min at 35,000×g. Supernatant was applied to a Ni-NTA column (GE Healthcare), which was then washed with 40 mM sodium phosphate buffer, 400 mM NaCl, 40 mM imidazole (pH 8.0), and eluted with 40 mM sodium phosphate buffer, 350 mM NaCl, 300 mM imidazole (pH 8.0). Eluted protein was diluted with 40 mM sodium phosphate buffer, 350 mM NaCl to an imidazole concentration of <40 mM and incubated with thrombin O/N at room temperature. Phenylmethylsulfonyl fluoride was then added to block further cleavage, and the protein solution was again applied to a Ni-NTA column. Even without the His_6_ tag, which was removed by thrombin cleavage, mimivirus R596-4C bound the Ni-NTA column at imidazole concentrations up to 40 mM. The thrombin-cleaved R596-4C construct was eluted with 40 mM sodium phosphate buffer, 400 mM NaCl, 40 mM imidazole (pH 8.0), and further purified by size exclusion in 20 mM Tris, 300 mM NaCl (pH 7.7). The major peak, corresponding to enzyme dimer, was collected and concentrated to ∼15 mg/ml as determined spectroscopically at 446 nm (ε = 11,300 M^−1^ cm^−1^) in 6 M guanidine-HCl containing 20 mM sodium phosphate buffer (pH 6.5). A selenomethionine variant was prepared according to published protocol [27] and purified as for wild-type enzyme.

### Crystallization, Data Collection, and Structure Refinement

Crystals of wild-type R596-4C and the selenomethionine variant were grown by hanging drop vapor diffusion over either of several well solutions. The first, which produced the crystals used for structure solution, contained 0.1 M HEPES (pH 7.0), 200 mM magnesium chloride, 200 mM lithium sulfate, 5% v/v 2-methyl-2,4-pentanediol (MPD), and 15–20% w/v PEG 1500. The second contained 0.1 M Bis-Tris (pH 6.0), 100 mM sodium citrate, 5–8% v/v dimethyl sulfoxide (DMSO), 9–15% v/v isopropanol, and 3% w/v PEG 2000 monomethyl ether (MME). In the latter cocktail, methanol could be substituted for the DMSO. R596-4C crystals in their respective mother liquors were mixed with an equal volume of a solution containing 50% v/v glycerol and 10% v/v MPD before flash-freezing in liquid nitrogen.

Diffraction data were collected on the ID29-1 beamline at the European Synchrotron Radiation Facility (ESRF), Grenoble, France, using a Pilatus 6 M detector. Native and selenomethionine data were integrated and scaled using XDS [28] and SCALA [29]. Heavy atom positions were located and phases were calculated using Phenix [30]. Model building and refinement were done using COOT [31] and CNS [32], respectively. The R596-4C structure has no Ramachandran outliers and scores in the 86^th^ percentile of structures of comparable resolution (2.21+/−0.25 Å) according to MolProbity [33].

### Enzyme Activity Assays

Sulfhydryl oxidase activity was measured at 25°C by monitoring oxygen consumption in a Clarke-type oxygen electrode (Hansatech Instruments Ltd.). R596-4C or R596-2C was diluted into 50 mM potassium phosphate buffer (pH 7.5), 300 mM NaCl, and 1 mM EDTA to obtain a volume of 990 µL, and reactions were initiated by injection of 10 µL of a 100X DTT stock to obtain the final desired DTT concentration. The enzyme concentration in the reaction mix was 1 µM.

### PDB Accession Code

The atomic coordinates and structure factors were deposited in the Protein Data Bank (www.rcsb.org) with PDB codes 3TD7.
